# Unlocking Prognostic Genes and Multi-Targeted Therapeutic Bioactives from Herbal Medicines to Combat Cancer-Associated Cachexia: A Transcriptomics and Network Pharmacology Approach

**DOI:** 10.3390/ijms25010156

**Published:** 2023-12-21

**Authors:** Subramanian Muthamil, Pandiyan Muthuramalingam, Hyun-Yong Kim, Hyun-Jun Jang, Ji-Hyo Lyu, Ung Cheol Shin, Younghoon Go, Seong-Hoon Park, Hee Gu Lee, Hyunsuk Shin, Jun Hong Park

**Affiliations:** 1 Herbal Medicine Resources Research Center, Korea Institute of Oriental Medicine, Naju 58245, Republic of Korea; muthamils@kiom.re.kr (S.M.); khy9514@kiom.re.kr (H.-Y.K.); hyunjun@kiom.re.kr (H.-J.J.); jhlyu@kiom.re.kr (J.-H.L.); ucshin@kiom.re.kr (U.C.S.); 2 Division of Horticultural Science, College of Agriculture and Life Sciences, Gyeongsang National University, Jinju 52725, Republic of Korea; pandianmuthuramalingam@gmail.com (P.M.); shinpomo@gnu.ac.kr (H.S.); 3 Korean Medicine (KM)-Application Center, Korea Institute of Oriental Medicine, Daegu 41062, Republic of Korea; gotra827@kiom.re.kr; 4 Genetic and Epigenetic Toxicology Research Group, Korea Institute of Toxicology, Daejeon 34141, Republic of Korea; seonghoon.park@kitox.re.kr; 5 Immunotherapy Research Center, Korea Research Institute of Bioscience and Biotechnology, Daejeon 34141, Republic of Korea; hglee@kribb.re.kr; 6 Korean Convergence Medicine Major, University of Science & Technology (UST), KIOM Campus, Daejeon 34054, Republic of Korea

**Keywords:** cancer-associated cachexia, herbal medicines, network pharmacology, phytocompounds, molecular docking, prognostic genes

## Abstract

Cachexia is a devastating fat tissue and muscle wasting syndrome associated with every major chronic illness, including cancer, chronic obstructive pulmonary disease, kidney disease, AIDS, and heart failure. Despite two decades of intense research, cachexia remains under-recognized by oncologists. While numerous drug candidates have been proposed for cachexia treatment, none have achieved clinical success. Only a few drugs are approved by the FDA for cachexia therapy, but a very low success rate is observed among patients. Currently, the identification of drugs from herbal medicines is a frontier research area for many diseases. In this milieu, network pharmacology, transcriptomics, cheminformatics, and molecular docking approaches were used to identify potential bioactive compounds from herbal medicines for the treatment of cancer-related cachexia. The network pharmacology approach is used to select the 32 unique genes from 238 genes involved in cachexia-related pathways, which are targeted by 34 phytocompounds identified from 12 different herbal medicines used for the treatment of muscle wasting in many countries. Gene expression profiling and functional enrichment analysis are applied to decipher the role of unique genes in cancer-associated cachexia pathways. In addition, the pharmacological properties and molecular interactions of the phytocompounds were analyzed to find the target compounds for cachexia therapy. Altogether, combined omics and network pharmacology approaches were used in the current study to untangle the complex prognostic genes involved in cachexia and phytocompounds with anti-cachectic efficacy. However, further functional and experimental validations are required to confirm the efficacy of these phytocompounds as commercial drug candidates for cancer-associated cachexia.

## 1. Introduction

Cachexia is defined as a combination of decreased food intake and increased body energy metabolism induced by tumor and host-derived factors [1]. Cachexia is an irreversible, multiorgan, and multifactorial syndrome that affects most of the human body, including adipose tissues, bones, intestines, brain, liver, and heart. Cachexia-related muscle wasting is common in patients with metastatic cancer, heart failure, kidney diseases, and chronic obstructive pulmonary diseases [2]. Generally, cachexia affects 50–80% of cancer patients and causes a mortality rate of up to 80% [3,4]. Cachexia is a wasting disorder characterized by weight loss of both adipose tissue and skeletal muscle, as well as inflammation and anorexia, which is a common feature of cancer patients and responsible for cancer-associated death [5]. Cachexia is a serious consequence of all chronic diseases but is underestimated. Generally, the prevalence of cachexia in pancreatic and gastric cancer patients is very high (87%); colon, lung, and prostate cancer types have a prevalence rate of 61%; and breast cancer, sarcoma, and leukemia have a 40% incidence of cachexia. Although many therapeutic approaches are recommended for cachexia, no direct treatment options are available yet. Although weight loss can occur in the early stages of cancer, treatment of cachexia has been started at the end stage of the disease; therefore, prevention of cachexia has not been the standard approach in clinical practices. In cachexia therapy, the drugs should target tumor-associated inflammation, increase anabolism, decrease catabolism, stimulate appetite, improve physical fitness, and achieve weight gain and muscle growth, thereby prompting the survival of patients and improving their quality of life [6,7,8]. As per the Food and Drug Administration, USA, the administration of human growth hormones and megestrol acetate can be used for cancer-associated cachexia (CAC) patients or AIDS-related cachexia. In addition, anamorelin, a ghrelin receptor agonist, was approved in Japan in 2020 for CAC, which significantly improved the lean body mass in cachexia patients with non-small cell lung cancer, pancreatic cancer, colorectal cancer, and gastric cancer; however, physical function and prognosis were not improved [9]. The international guidelines for the management of CAC patients recommend a combination of nutritional, physiological, and pharmacological interventions for cachectic therapy to improve patients’ quality of life [5]. Further, it is important to increase awareness of CAC and start a combination of interventions in cachexia or pre-cachexia patients to improve their skeletal muscle function. With this background, there is an immediate need for new therapeutic approaches for cachexia syndrome that involve oncologists, dietitians, physicians, and radiologists.

Medicinal plants are the sources of pharmaceutical agents used for the treatment of various diseases among human populations. Globally, bioactive compounds from plants, crude extracts, or herbal formulations have been used for pharmacotherapeutic purposes for a long time [10,11]. In addition, herbal medicines are reported for their pharmacological activities, including anti-microbial, anti-cancer, anti-inflammatory, anti-mutagenic, antioxidant, analgesic, immune-modulatory, and anti-depressant activities [12]. Although medicinal plants are reported for their various biological and pharmacological activities, reports related to cachexia therapy using herbal medicines are very limited. For instance, curcumin from *Curcuma longa* has already been reported for its anti-inflammatory, antioxidant, and tumoricidal properties, which also stimulate anti-cachectic effects in atrophic C2C12 muscle cells [13,14,15]. In the aspect of traditional herbal medicines, named “Hozai” prescriptions consist of *Panax ginseng*, *Ziziphus jujuba* Mill., *Wolfiporia extensa*, and *Glycyrrhiza glabra* L. used in East Asia for underlying diseases or cachexia. Likewise, Russian traditional herbs *Aralia mandshurica* Rupr. et Maxim., *Echinopanax elatum* Nakai, *Eleutherococcus senticosus* (Rupr. & Maxim.) Maxim., *Leuzea carthamoides* (Willd.) DC., *P. ginseng* C.A.Mey., *Schisandra chinensis* (Turcz.) Baill., *Rhodiola rosea* L., and *Sterculia platanifolia* were used for the muscle wasting syndrome [16]. In addition to that, plant-derived antioxidants [17], flavonoids [18,19], and anti-cytokines [20] are also used for CAC therapy. Although the recent literature survey reported several phytocompounds and/or herbal medicines for cachexia therapy [16,17,18,19,20,21,22], targeting genes/pathways, molecular interactions, and the mode of action of the phytocompounds remain unclear.

In this milieu, network pharmacology is a promising approach used for the understanding of molecular targets of phytocompounds in recent years. Network pharmacology is a trending method used to integrate computational methods and clinical omics data to explore the pharmacokinetic mechanisms of traditional medicines and their bioactive compounds [23]. In addition, network pharmacology is the combined in silico method for establishing a compound/peptide-target gene/disease network to unravel the mechanism of action of multiple bioactive leads from traditional medicines [24]. Application of this modern-era approach to traditional/conventional medicines is an evolving approach for the identification of bioactive compounds and their putative molecular targets. This method includes the following steps: (i) Data mining; (ii) construction of networks and analysis; and (iii) validation of results (in silico, in vitro, and in vivo) [25,26]. In recent years, this method has been used for the identification of target bioactive compounds from plants against several life-threatening diseases, such as COVID-19 [10,27], lung cancer [26,28], cervical cancer [29], oral cancer [30], and neurological diseases [31,32]. Even though this promising network pharmacology approach explores traditional herbal medicines against several cancer types and neurological diseases, the application of this method to identify specific targets against muscle-wasting cachexia remains less understood. Therefore, the present study aims to identify novel bioactive compounds from traditional herbal medicines against CAC and its molecular pathways using transcriptomics profiling and network pharmacology approaches. The identification of specific targets from medicinal plants might be a breakthrough in cachexia research, and this study could pave the way for the identification of plant-based drugs to combat CAC and other diseases.

## 2. Results

### 2.1. Selection of Phytocompounds

A total of 34 phytocompounds were selected from the different herbal medicines used for muscle wasting. The list of selected herbal medicines and phytocompounds is listed in Table 1. The canonical SMILES of the phytocompounds were retrieved from the PubChem database and given in Supplementary Table S1.

### 2.2. Selection of Unique CAC Genes and Human Targets

Direct human targets of phytocompounds derived from herbal medicines were predicted using the SwissTargetPrediction Tool. Based on the results of SwissTargetPrediction, 32 out of 237 genes were identified as unique curated genes. The identified unique genes with their respective phytocompounds are listed in Table 2. Among the selected phytocompounds, KFR, CFA, GST, AGS, AGT, JJA, CGA, and XNS targeted a high number of unique genes related to CAC.

### 2.3. Molecular Network of Human Targets and Phytocompounds

**Figure 1:**
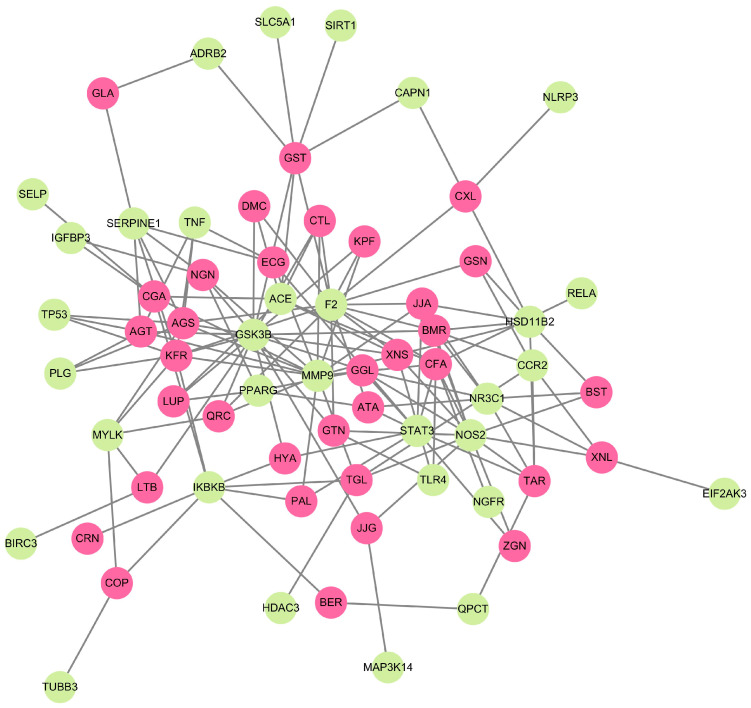
The CTN represents the interaction between phytocompounds from herbal medicines and CAC-related target genes. Green and pink colors indicate the gene targets and phytocompounds, respectively.

Compound-target network (CTN) was depicted using Cytoscape v3.10.0 (Figure 1). It was observed that 34 selected phytocompounds were closely connected with the 32 unique CAC genes. This result highlights the multiple targets of selected phytocompounds. Also, the interaction of these phytocompounds proves that they might be used as potent drug targets for CAC.

### 2.4. Protein Interaction and Pathway Analysis

**Figure 2:**
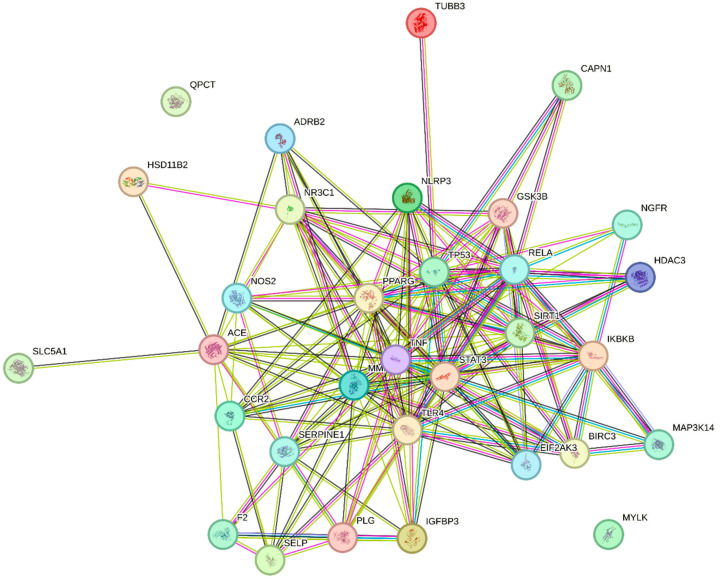
Protein–protein interaction network of CAC-associated genes. Lines represent interactions between genes, and nodes represent the protein partners of respective genes. Each color of the nodes represents the interacting evidence between the proteins. Pink color—experimentally determined/post-translational modifications; blue color—gene co-occurrence; green color—gene neighborhood; black color—co-expression; red color—gene fusion. Nodes with ribbon-like structures represent the availability of protein 3D structural information that is predicted.

**Figure 3:**
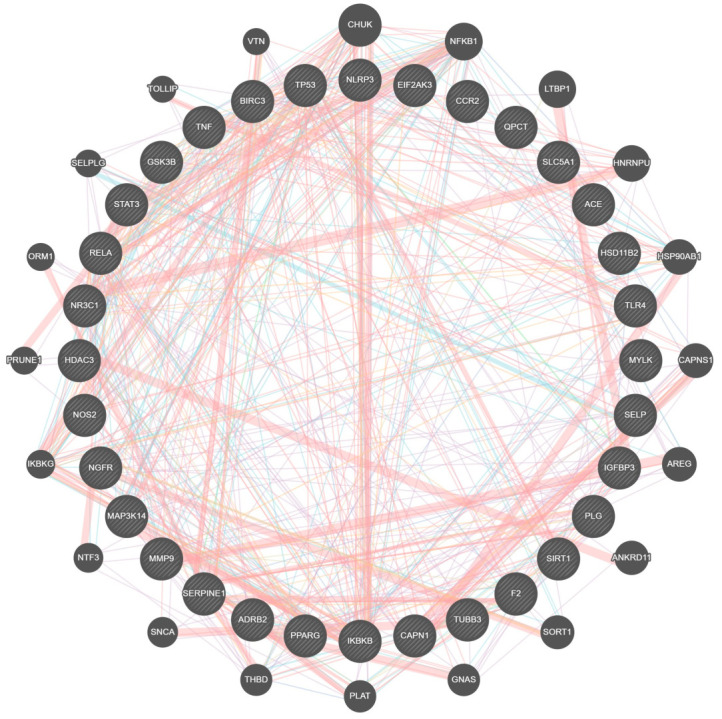
Visualization of CAC-related genes and their functional cross-talks. Colored lines between the genes indicate several types of interacting evidence. Orange—physical interaction; periwinkle—co-expression; sky blue—involvement in pathways; green-genetic interaction; blue color—localization.

Figure 2 illustrates the complex protein–protein interaction (PPI) of the unique genes in CAC. The interaction analysis shows a higher number of interactions between genes involved in cachexia pathways, including adipogenesis, signal transduction, cellular senescence, etc., as performed by the STRING v12.0 database. The molecular cross-talks had 32 nodes and 168 edges, and the average nodal degree of closely related proteins was 10.5 (Figure 2). The PPI enrichment *p*-value of these CAC-related genes is <0.01. In addition, functional interactions between 32 unique genes were analyzed using GeneMANIA based on the integrated human genomics and transcriptomics data (Figure 3). These cross-talks represent several types of interacting evidence, such as genetic interactions, localization, physical interactions, involvement in pathways, and co-expression.

### 2.5. Gene Ontology Enrichment Analysis

**Figure 4:**
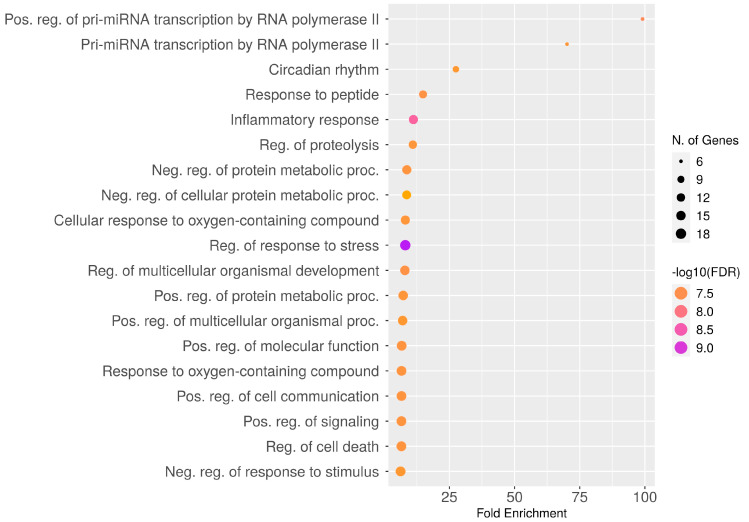
Dot plot of GO enrichments of CAC-associated unique genes with their biological processes. The number of unique genes falling in each GO biological process term is directly proportional to the count ball size. Based on the level of significance (-log10(FDR)) in enrichment, the balls are color-shaded.

**Figure 5:**
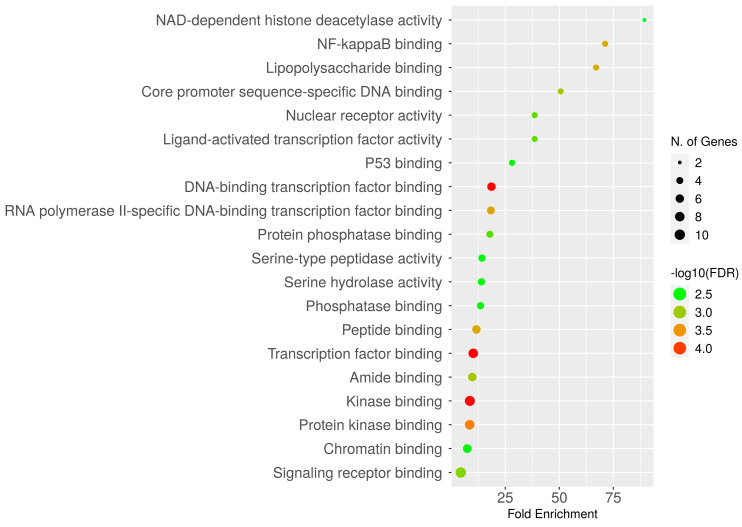
Dot plot of GO enrichments of CAC-associated unique genes with their molecular function. The number of unique genes falling in each GO molecular function term is directly proportional to the count ball size. Based on the level of significance (-log10(FDR)) in enrichment, the balls are color shaded.

To explore the biological processes and molecular functions of unique genes involved in CAC, GO enrichment analysis was performed. In the aspect of biological functions, the target genes are involved in the regulation of proteolysis, circadian rhythm, cell death, stress response, inflammatory response, cellular response to oxygen-containing compounds, etc. (Figure 4). In addition, the genes are involved in the positive and negative regulation of protein metabolism. Furthermore, unique genes are enriched in numerous molecular functions, including signaling receptor binding, chromatin binding, transcription factor binding, peptide binding, several enzymatic binding activities, etc. (Figure 5).

### 2.6. Over-Representation Analysis (ORA)

**Figure 6:**
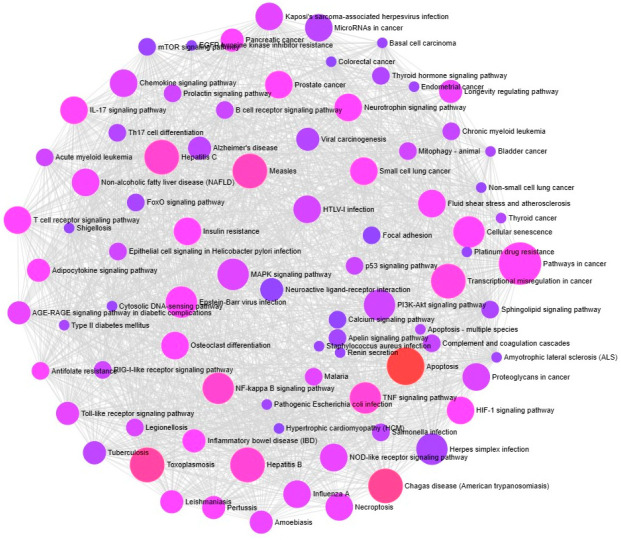
ORA enrichment analysis. Involvement of unique genes in metabolic and signaling pathways associated with cancer and other cachexia-related diseases.

**Figure 7:**
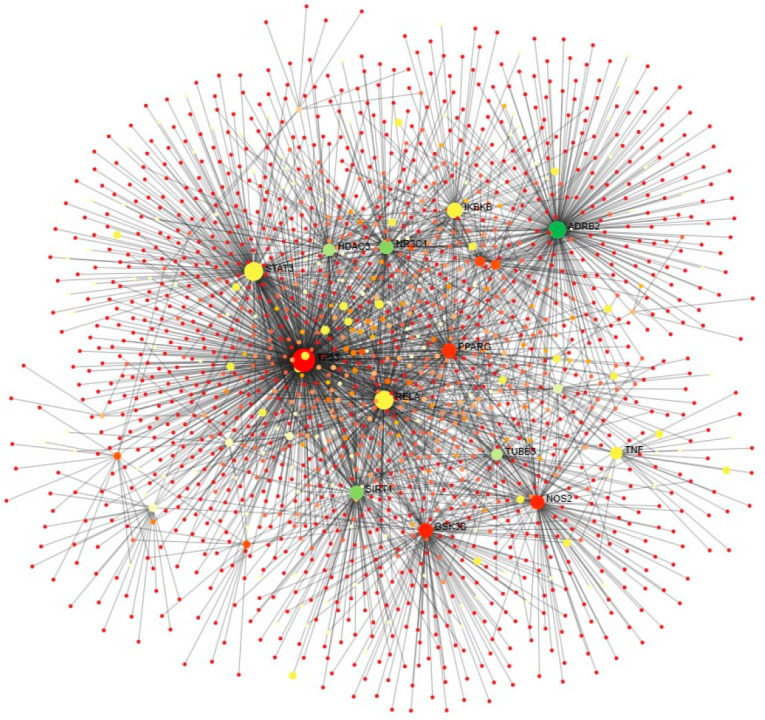
PPI is related to adipocytokine signaling. Involvement of unique genes in adipose tissue-specific signaling pathway and the representing nodes are highlighted in yellow.

ExpressAnalyst 3.0. results confirm that these unique genes are involved in CAC-related processes, including multiple cancer pathways (small and non-small cell lung cancer, pancreatic colorectal, endometrial, prostate cancer, bladder cancer, thyroid), signaling pathways (MAPK signaling, P13K-Akt signaling, p53 signaling, tumor necrosis factor (TNF) signaling, nuclear factor-kappa B (NF-κB) signaling, toll-like receptor (TLR) signaling, interleukins (IL)-17 signaling, and T-cell receptor signaling), apoptosis, and cellular senescence. Also, the genes involved in other viral, bacterial, parasite-related diseases, diabetes, and Alzheimer’s diseases (Figure 6). Further, the Network Analyst online tool is used to identify the tissue-specific involvement of unique genes in adipocytokine signaling, considered one of the important CAC-related molecular pathways (Figure 7). These results confirm the role of unique genes in cachexia metabolic pathways. Phytocompounds targeting these players could be a potential drug target for CAC.

### 2.7. KEGG Pathway Enrichment Analysis

**Figure 8:**
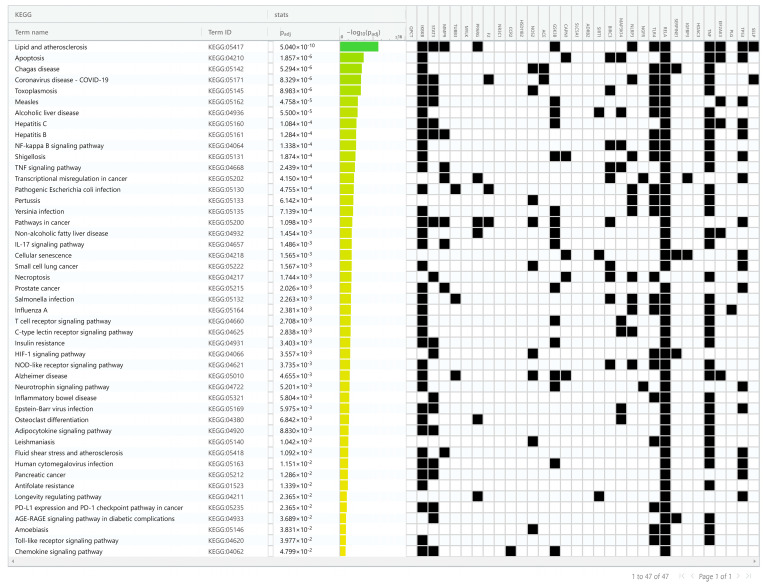
Biological pathway enrichment analysis of identified bioactives against CAC. The ordinate displays the −log10 (adjusted *p*-value) of the KEGG terms and the enrichment pathway analysis identification result according to an adjusted *p*-value of 0.05.

KEGG (Kyoto Encyclopedia of Genes and Genomes) pathway analysis revealed that unique genes were involved in 47 different KEGG pathways (Figure 8 and Supplementary Table S2) based on an adjusted *p*-value of 0.05. Overall, the unique genes are mainly involved in lipid and atherosclerosis, apoptosis, NF-κB signaling, TNF signaling, cancer pathways, and other signaling pathways related to CAC. Among the unique genes, TNF, RELA, IKB3B, STAT3, TLR4, TP53, and GSK3B are the major players intricate in many of the identified KEGG pathways.

### 2.8. Unique Genes Survival Analysis

**Figure 9:**
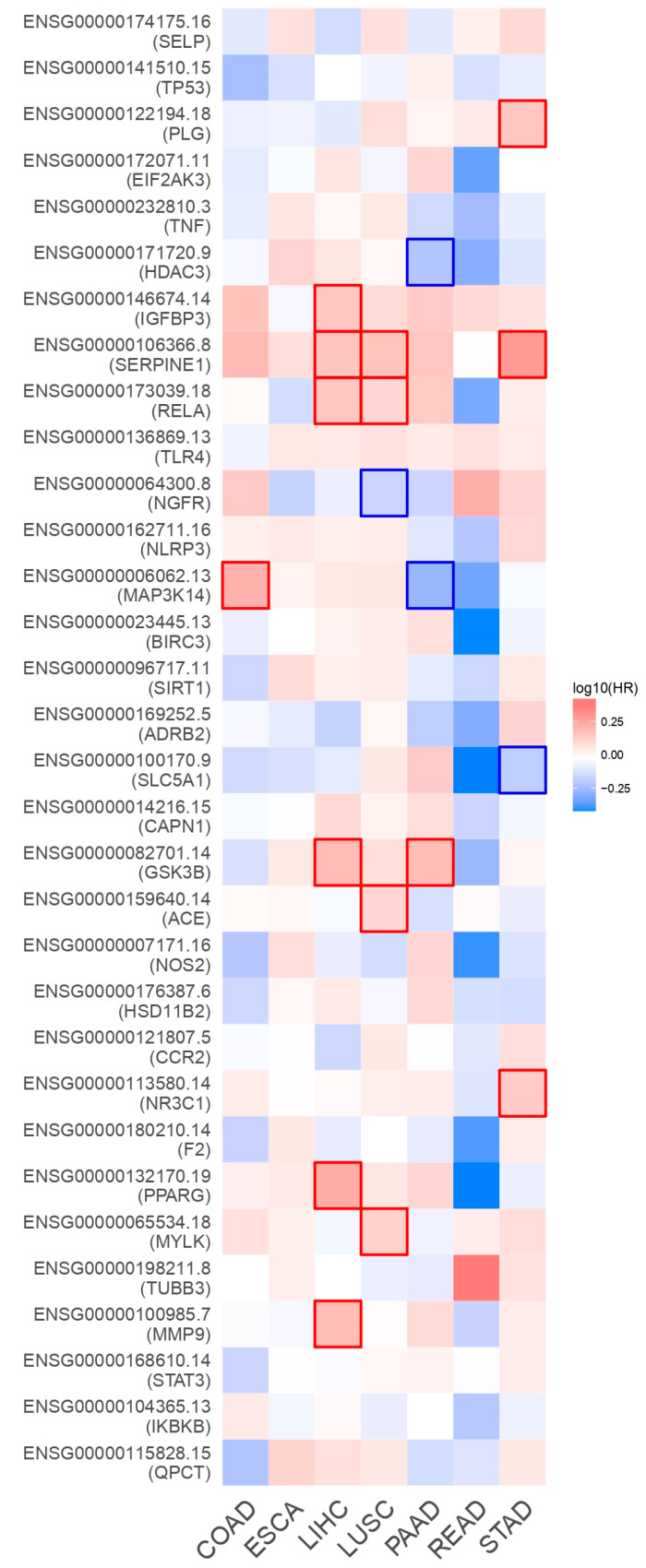
The survival heatmap highlights the prognostic impacts of unique gene expression levels based on the COAD, ESCA, LIHC, LUSC, PAAD, READ, and STAD datasets. The heatmap emphasizes the hazard ratios on log10 scale for the CAC-associated genes. Red and blue colors denote higher and lower risks, respectively. The darkened rectangular frames show significant favorable and unfavorable results in prognostic analyses.

Unique genes survival map analysis was performed using the gene expression levels of various cancer datasets obtained from gene expression profiling interactive analysis server 2 (GEPIA2). The results of survival analysis are represented as a heatmap (Figure 9) to explore the functions of CAC-related genes in multiple cancer types, including colon adenocarcinoma (COAD), esophageal carcinoma (ESCA), liver hepatocellular carcinoma (LIHC), lung squamous cell carcinoma (LUSC), pancreatic adenocarcinoma (PAAD), rectum adenocarcinoma (READ), and stomach adenocarcinoma (STAD). It was observed that, among the unique genes, the SERPINE1 gene is significantly expressed in STAD, LIHC, and LUSC; RELA showed a higher level of expression in LIHC and LUSC; and GSK3B exhibited increased expression in LIHC and PAAD. Other genes such as PPARG, PLG, IGFBP3, MAPK3K14, ACE, NR3C1, MYLK, and MMP also exhibited considerable expression in different cancer types. These results uncovered that significantly expressed genes are identified as prognostic genes, which are key players in reducing the survival rate of CAC patients.

### 2.9. Pharmacological Properties of Phytocompounds

Pharmacological characteristics (GPCR, KI, Ncr, Pi, Ei, and nVio) of selected phytocompounds were determined and listed in Table 3. Significant phytocompounds were selected based on the enzyme inhibitor activity score (>0.5) and nVio values. Accordingly, the phytocompounds berberine, palmatine, coptisine, lupeol, ginsenosides, jujubogenin, beta-sitosterol, beta-amyrin, xanthinosin, and chlorogenic acid exhibited high Ei scores. In addition, 14 phytocompounds have an nVio value of zero (Table 3).

### 2.10. Molecular Docking Analysis

**Figure 10:**
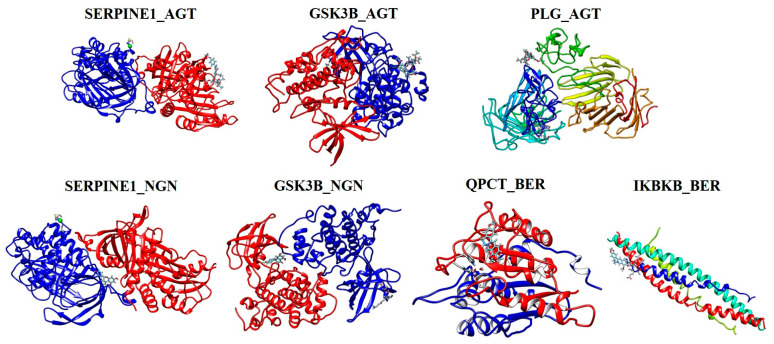
Three-dimensional representation of molecular docking analysis. Interacting profile of phytocompounds AGT, NGN, and BER and the targets SERPINE1, GSK3B, PLG, QPCT, and IKBKB.

Based on the results obtained from survival heatmap analysis, unique genes such as SERPINE1, GSK3B, QPCT, IKBKB, and PLG were selected as macromolecules (proteins) for molecular docking studies. Phytocompounds, namely NGN, AGT, and BER, target the selected unique genes and were used as ligands in SwissDock analysis. The molecular docking results of selected phytocompounds and genes are illustrated in Figure 10, and the binding scores are listed in Table 4. Among the selected phytocompounds, AGT binds with SERPINE1, GSK3B, and PLG genes with the highest binding energies of −8.54, −9.36, and −8.14 kcal/mol, respectively. Comparatively, NGN interacts with SERPINE1 and GSK3B with binding scores of −7.02 and −7.04 kcal/mol, respectively.

### 2.11. Effect of the Bioactive Compounds on 3T3-L1 Cell Viability

**Figure 11:**
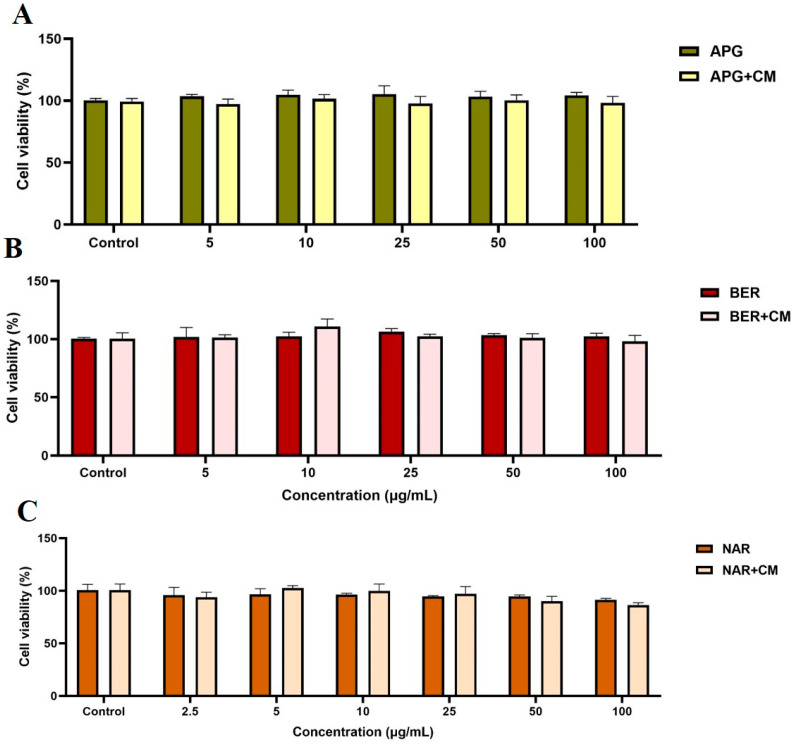
Cytotoxicity effect of phytocompounds. APG (**A**), BER (**B**), and NGN (**C**) on cell viability of preadipocyte 3T3-L1 under cachectic condition.

The cytotoxic effects of apigenin (APG), BER, and NGN on preadipocyte 3T3-L1 cell viability under cachectic conditions were assessed. Both the phytocompounds did not affect the 3T3-L1 cell viability up to 100 µg/mL concentration. Also, the compounds have no cytotoxic effect up to 50 µg/mL concentration, even under cachectic conditions (Figure 11). These results prove that the phytocompounds APG, BER, and NGN might be a drug choice for cachexia therapy.

## 3. Discussion

Globally, cancer is one of the leading causes of morbidity and mortality. Cachexia replaced the term “malnutrition”, particularly in older cancer patients (>70 years), which leads to poor treatment response, frequent infections, increased treatment toxicity, and lower survival rates [59]. Generally, successful or unsuccessful anti-cancer treatment leads to an increased rate of catabolism and cachexia symptoms [60]. Due to the increased prevalence of cachexia in cancer patients, various national and international medical societies, including the American Society of Clinical Oncology (ASCO), the Global Leadership Initiative on Malnutrition (GLIM), the European Society for Medical Oncology (ESMO), and the European Society for Clinical Nutrition and Metabolism (ESPEN), have published guidelines and recommendations for managing CAC and improving nutrition [61]. CAC remains an underdiagnosed complex syndrome that requires multimodal or combination therapy, including nutritional intervention and physical exercise, psychological counseling, and pharmacological intervention. In cachectic patients with inadequate food intake, nutritional interventions such as dietary counseling, oral nutritional supplements, tube feeding, and parenteral nutrition are required. In recent years, reports suggest that physical exercise is beneficial for cancer patients. Although it does not have an impact on CAC patients, multimodal therapy with physical activity improves physical endurance and depression [62,63]. In the case of pharmacological interventions, many drugs have been investigated in clinical trials for the treatment of CAC, such as corticosteroids, progestins, cannabinoids, androgens, olanzapine, prokinetics, ghrelin receptor agonists, and non-steroidal anti-inflammatory drugs. Among the drugs used in cachexia therapy, only corticosteroids and progestins exhibited beneficial effects on inducing appetite and/or body weight, while the output of other drug agents was disappointing [64]. Even though megestrol acetate is approved by the FDA for the treatment of anorexia and cachexia-related weight loss in AIDS patients, human participants treated with this drug have a small effect on appetite and weight gain; megestrol acetate does not improve the quality of life; and some adverse effects are frequent in the patients [9,65,66]. There are several barriers associated with cachexia treatment, such as limited awareness among oncologists of the impact of cachexia, terminological confusion between cachexia, malnutrition, sarcopenia, and the complexity of the disease, limited evidence for high-quality therapeutic intervention, and the patient’s mental state [61]. Until now, there has been no standardized treatment option for CAC, and the complexity of this syndrome presents an open challenge to oncologists and researchers in the aspect of discovering new therapeutic approaches. Therefore, understanding the mechanism of CAC is the need of the hour to discover new promising therapeutic approaches using herbal medicines or formulations instead of drugs.

Since ancient times, traditional herbal medicines have been used for the treatment of various diseases, and recent literature has gradually revealed the therapeutic mechanisms of bioactive compounds/phytocompounds [21]. Worldwide, plant-based pharmaceuticals and nutraceuticals have gained much attention due to their efficacy, safety, bioavailability, and few or no side effects [67]. Hypothetically, suppression of anti-inflammatory cytokines such as TNF-α, IL-1, and IL-6 may lead to an improvement in CAC. In traditional medicinal systems, fenugreek and chicory have been demonstrated for their anti-inflammatory potential [68,69]. Additionally, Park et al. (2019) reported that fourteen herbal medicines and their phytocompounds suppressed CAC symptoms through anti-inflammation effects, modulation of the ubiquitin-proteasome system, and regulation of the neuroendocrine pathway [70]. In addition, recent evidence has revealed the anti-cachectic potential of phytocompounds such as berberine [71], curcumin [15], resveratrol [72], quercetin [73], and epigallocatechin-3-gallate [74,75], which consist of different mechanisms of action. In addition, nutritional supplements such as creatine, eicosapentaenoic acid, insulin, and beta-adrenergic agonist adenosine triphosphate (ATP) are also used for the control and management of CAC [67]. Although many pharmacological and nutritional interventions have been reported for CAC, many of them failed in clinical trials and succeeded only in animal models. Also, the molecular mechanism and mode of action of many phytocompounds or herbal formulations remain poorly understood.

With this background, network pharmacology combined with bioinformatics, systems biology, and pharmacology is a trending research field in cancer research. Meanwhile, the reports related to the implementation of this network pharmacology approach in cachexia research are limited. To the best of our knowledge, the present study is a first attempt to identify the potential anti-cachectic phytocompounds from herbal medicines based on their molecular targets using a network pharmacology approach. For this purpose, 34 phytocompounds from 12 different herbal medicines and 253 genes were selected based on the previous literature used in this study. Among the selected genes, 32 genes directly interact with the selected phytocompounds and are recognized as unique genes. Based on our previous literature review, cachexia is a complex syndrome connected with several signaling pathways, including TGF-β signaling activation, PI3K/Akt/mTOR signaling, the renin–angiotensin–aldosterone system (RAAS) pathway, myostatin and activin signaling, and the hypoxia/HIF-1 pathway [4]. So, the anti-cachectic drugs should target multiple genes/pathways that could provide successful results. In this study, the phytocompounds KFR (10 genes), CFA, GST (8 genes), XNS, CGA, JJA, AGS, and AGT (7 genes) target a maximum number of unique genes involved in the CAC pathway. Correspondingly, CTN analysis results confirm that each phytocompound targets multiple genes involved in different CAC pathways. Therefore, the selected phytocompounds have therapeutic potential against CAC, and these compounds can be used for multimodal cachexia therapy.

The results of PPI analysis confirm the interaction between CAC pathways, including the inflammasome pathway (NLRP3, NOS2, SIRT1, TLR4, STAT3, IGFB3), adipogenesis and insulin resistance (PPARG, GSK3B), pathogenic signaling (SERPINE1), cell cycle and apoptosis (SIRT1, TNF), and tissue remodeling (PLG, MMP9) [76]. Also, GeneMANIA results confirm the signaling cross-talks between genes as interaction evidence; as shown in the results (Figure 3), the unique genes involved in multiple pathways related to CAC. In general, muscle wasting is related to several biological processes, including respiration, the balance between protein synthesis and degradation, energy metabolism, and glucose homeostasis. Moreover, autophagy, ubiquitin-mediated proteasome degradation, and lipolysis are the major molecular mechanisms underlying skeletal muscle atrophy [77]. Further, gene ontology enrichment analysis reveals the involvement of unique genes in the various biological processes and molecular functions related to CAC. In particular, the genes are mainly involved in the inflammatory response, regulation of cell death and proteolysis, and positive and negative regulation of the protein metabolic process (Figure 4A).

In this study, for the first time, we have used ExpressAnalyst and NetworkAnalyst platforms to create an enrichment network of unique genes involved in CAC. The obtained results confirm that the selected unique genes relate to multiple cancer pathways. Additionally, adipose tissue-specific PPI also highlights the interaction between unique genes such as STAT3, IKBKB, RELA, and TNF. The STAT3 gene is highly expressed in the adipose tissues of mice and humans, which promotes adipogenesis by activating stem cell proliferation during the early stage of differentiation. Although the role of STAT3 is still not clear in adipogenesis [78]. Equally, IKBKB encodes serine kinases and inhibits NF-κB signaling, which plays a key role in the inflammatory response, immune cell proliferation, and metabolic diseases [79]. Conversely, RELA is a subunit of the NF-κB family that is downregulated by rapamycin and inhibits adipogenic differentiation in murine mesenchymal stem cells [80]. Similarly, TNF-α inhibits adipogenesis and bone formation by activating the Wnt-signaling pathway in pre-adipocytes and osteoclasts, respectively [81]. KEGG pathway functional enrichment analysis represents the role of unique genes in the various signaling pathways and immune responses associated with CAC. The results of the survival analysis highlight the significant gene expression of a few prognostic genes. These prognostic genes are also known as unfavorable genes because their expression may reduce the overall survival of CAC patients [26,82]. Phytocompounds, or drugs mainly targeting these prognostic genes, might be useful for cachexia therapy.

According to Poisson et al. (2021), the prevalence of cachexia in gastrointestinal tract cancers is 76.4%, which includes liver, pancreatic, esophageal, and gastric cancers [59]. In survival heat map analysis, the SERPINE1 gene exhibited significant upregulation in STAD, LIHC, and LUSC. In line with this, the SERPINE1 gene is reported as one of the regulated genes during TGF-β-induced osteogenic and adipogenic differentiation in human mesenchymal stem cells [83]. Similarly, RELA and GSK3B genes are known to be involved in adipogenic differentiation [80,84]. As stated earlier, these genes are recognized as prognostic genes or unfavorable genes, which reduce the survival of different types of cancer patients and also decrease the survival rate of CAC patients. Besides, the pharmacological properties of phytocompounds such as GPCR, Pi, Ki, Ncr, Ei, and nVio were measured according to the rule-of-five (Ro5) drug discovery [85]. These pharmacological features are important for selecting “drug-like” properties, including oral bioavailability, solubility, and permeability of the drug. Based on the survival analysis and pharmacological properties, the phytocompounds AGT, BER, and NGN and the genes SERPINE1, GSK3B, QPCT, IKBKB, and PLG were selected for docking analysis. The selected phytocompounds, AGT, BER, and NGN, bind with the SERPINE1, GSK3B, QPCT, IKBKB, and PLG genes with the highest binding energy (>−7.0). These results confirmed that the phytocompounds significantly interact with the prognostic genes and their related signaling pathways.

Apigetrin, or apigenin-7-O-beta-D-glucoside, a dietary flavonoid found in many plants, has already been reported for its anti-inflammatory, anti-cancer, anti-mutagenic, antioxidant, and anti-diabetic effects. Also, the compound inhibits the cell proliferation of human hepatocellular cancer cells (Hep3B) by inducing apoptosis and necrosis; thereby, this compound is suggested for the treatment of liver cancer [86]. Although the compounds significantly inhibited lipid accumulation, they did not affect the cell viability of 3T3-L1 preadipocytes at a 100 µM concentration [87]. Similarly, berberine is an alkaloid found in many plants, including the *Berberis* genus, traditionally used for the treatment of diabetes, constipation, inflammatory disorders, and infectious diseases [88]. In mice with colon cancer cells (HCT116), berberine decreased the hallmarks of CAC, including intestinal mucosal damage, systemic inflammation, and a reduction in food intake [71]. In the same way, naringenin is a colorless, flavorless flavonoid in edible fruits that has several pharmacological properties such as anti-mutagenic, anti-inflammatory, anti-cancer, hepatoprotective, anti-atherogenic, and antimicrobial properties [89,90]. In the current study, the cytotoxic effects of phytocompounds on 3T3-L1 preadipocytes were assessed using the cell viability assay. The results confirmed that even at higher concentrations, the phytocompounds have no cytotoxic effect on 3T3L1 cells. Also, the phytocompounds rescue the preadipocyte cells from apoptosis under cachectic conditions. Overall, the phytocompounds AGT, BER, and NGN from *C. morifolium* Ramat., *C. chinensis,* and *C. lacryma-jobi* were identified as target herbal medicines for CAC therapy. The phytocompounds AGT, BER, and NGN target the genes SERPINE1, GSK3B, IKBKB, QPCT, and PLG involved in molecular pathways related to CAC.

## 4. Materials and Methods

### 4.1. Screening of Herbal Medicines and Phytocompounds

Based on the previous literature available for cachexia, ten different medicinal plants were selected for this study [16,21,44]. Plants such as *C. chinensis*, *A. macrocephala*, *P. ginseng* C. A. Meyer, *Z. jujuba*, *C. lacryma-jobi*, *T. foenum-graecum* L., *P. sativum*, *Z. officinale*, *A. japonica* Thunb., *X. strumarium* L., *C. morifolium* Ramatuelle, and *C. tinctorius* L. are used as traditional herbal medicines in different parts of the world, including Korea, China, Russia, and India [21,91]. Phytocompounds are the constituents of medicinal plants responsible for their bioactive properties. Phytocompounds from selected medicinal plants were identified through a literature survey and web sources.

### 4.2. Retrieval of Phytocompounds and Pharmacological Properties

A total of 34 compounds were selected for this study, and their canonical SMILES were collected from the PubChem database (https://pubchem.ncbi.nlm.nih.gov/ accessed on 16 August 2023). Canonical SMILES of selected phytocompounds were analyzed in the Molinspiration tool (https://www.molinspiration.com/ accessed on 18 August 2023) to identify their pharmacological properties, including G-protein coupled and nuclear receptor (GPCR, Ncr), enzyme inhibitor activity (Ki, Pi, and Ei), and number of violations (nVio). Phytocompounds/biomolecules violating more than one of Lipinski’s five rules [85] may have problems with bioavailability and not be considered potent drug molecules [32].

### 4.3. Checking of Human Targets and Curation of Unique Genes

The SwissTargetPrediction tool was used to determine the human targets of selected phytocompounds, and canonical SMILES were given as an input (www.swisstargetprediction.ch/, accessed on 21 August 2023) [92]. In addition, 238 reported cachexia-associated genes were collected through a literature survey and web search for manual comparative analysis to find the unique genes [93,94,95,96]. The list of cachexia-associated genes collected is given in Supplementary Table S3.

### 4.4. Compound-Target Network (CTN) Analysis

CTN analysis was built using the Cytoscape v3.10.0 open-source software plugin [97,98], which is used to construct phytocompound interactions with selected unique genes involved in CAC-related molecular pathways. In this interactive network, phytocompounds and related proteins/genes were represented as nodes, and their interactions were represented as edges/links.

### 4.5. Interactome and Gene Enrichment Analyses

The PPI network was constructed for the unique genes involved in CAC using the STRING v12.0 (string-db.org/v12.0/ accessed on 29 August 2023) [96] database with a high confidence interaction score of 0.7. This PPI network is used to understand the physical interactions and regulatory mechanisms of CAC-related genes. In addition, to understand the functional interactions between the unique target genes, GeneMANIA (https://genemania.org/ accessed on 1 September 2023) [99] analysis was performed. Further, Gene Ontology (GO) enrichment analysis of these unique CAC-associated genes was also performed using ShinyGO v0.77 (http://bioinformatics.sdstate.edu/go/ accessed on 1 September 2023) [100]. Important parameter threshold values were set as follows: FDR cutoff of 0.05 and −log10(FDR)-based color enrichment to obtain the GO biological process and molecular functions from the enriched GO terms. 

### 4.6. Over-Representation Analysis (ORA)

ORA is one of the popular approaches used in bioinformatics studies of omics datasets. ORA is used to statistically analyze the overrepresented genes in the obtained datasets [101]. For this purpose, the ExpressAnalyst tool (https://www.expressanalyst.ca/ExpressAnalyst/home.xhtml accessed on 4 September 2023) was used to check the involvement of the obtained genes in many CAC-related pathways. Further, the selected unique genes were imported into the Network Analyst 3.0. database (https://www.networkanalyst.ca/ accessed on 7 September 2023) for imputing the adipose tissue-specific CAC-related pathway analysis by the inbuilt KEGG [26,102].

### 4.7. KEGG Pathway Enrichment Analysis

Biological pathways enrichment (KEGG) analysis of unique genes involved in CAC-related pathways was studied through g:Profiler (https://biit.cs.ut.ee/gprofiler/gost, accessed on 11 September 2023) [103] against “*Homo sapiens*”. The KEGG term ID, consisting of an adjusted *p*-value < 0.05, was taken as significant. Further, the KEGG pathway terms were generated by an adjusted significant *p*-value from low to high.

### 4.8. Overall Survival Heat MAP Analysis

Overall survival and survival heat map analysis of unique genes were confirmed against COAD, ESCA, LIHC, LUSC, PAAD, READ, and STAD in-built datasets by the GEPIA2 server (http://gepia.cancer-pku.cn/ accessed on 14 September 2023) [104]. The GEPIA 2 was used to envisage the effect of the patient’s survival time based on cancer and normal samples from the Cancer Genome Atlas Program (TCGA) and the Genotype Tissue Expression Project (GTEx) databases. The survival heatmap map analysis of unique genes was imputed against COAD, ESCA, LIHC, LUSC, PAAD, READ, and STAD datasets with various parameters such as *p*-value FDR adjustment, survival time units in months, and significance level of 0.05.

### 4.9. Molecular Docking

Molecular docking is a computational method used to predict the binding affinity of phytocompounds (ligands) to their druggable targets (genes/proteins) [105]. Based on the prevalence of CAC in different cancer types and the data obtained from overall survival analysis, highly expressed unique genes in STAD and PAAD were selected for molecular docking [8,24]. A molecular docking study was performed using SwissDock (http://www.swissdock.ch/ accessed on 22 September 2023) [106]. The structure/ID of the phytocompound and CAC-related genes with their respective protein 3D structures were retrieved from the ZINC20 database [107] and protein data bank (PDB), respectively.

### 4.10. Cell Culture and Cachectic Condition Media

In the present study, mouse embryo 3T3-L1 preadipocytes (ATCC) were used. The 3T3-L1 cells were cultured in DMEM supplemented with 1% penicillin-streptomycin (P/S) and 10% bovine calf serum (BS). Then, the cells were incubated at 37 °C with 5% CO_2_ to reach optimum confluency. To prepare cachectic conditioned media (CCM), CT26 cells were seeded in a T175 flask (1 × 10^5^ cells/flask) with RPMI media consisting of 1% P/S and 10% fetal bovine serum (FBS) (Gibco; Grand Island, NY, USA) and incubated for 12 h [15]. Then, the RPMI medium was replaced with 30 mL of DMEM with 10% FBS and 1% P/S and incubated for 4 days. After incubation, 50 mL of fresh DMEM was added to the supernatants. After another 3 days, supernatants were collected by centrifugation (2000 rpm for 10 min at 4 °C) and filtered through a 0.22 μm cellulose acetate filter (Corning; Corning, NY, USA).

### 4.11. Cell Viability Assay

To assess the effect of APG, BER, and NGN on 3T3-L1 cell proliferation under cachectic conditions, a cell counting kit (CCK-8 kit; Sigma-Aldrich, St. Louis, MO, USA) was used [108]. 3T3-L1 cells were seeded (3 × 10^3^ cells/well) in 96-well plates and treated with various concentrations of APG, BER, and NGN (5 µg/mL to 100 µg/mL) and incubated at 37 °C for 48 h. After incubation, 10 µL of CCK-8 reagent was added to each well and incubated for 2 h. Then, the absorbance was measured at 450 nm using SpectraMax i3X (Molecular Devices, San Jose, CA, USA). The experiments were performed at least twice in independent experiments in triplicate to confirm reproducibility, and the data are represented as the mean ± SEM. Another name for apigetrin is apigenin 7-glucoside (https://pubchem.ncbi.nlm.nih.gov/compound/5280704 accessed on 25 September 2023). Therefore, in this experiment, apigenin was used.

## 5. Conclusions

The study revealed that phytocompounds from traditional herbal medicines used in many countries target the prognostic genes involved in cachexia-related pathways. As far as we know, this is the first study that employs integrated network pharmacology, omics profiling, gene, and functional enrichment analysis to find the key mechanisms underlying CAC. Also, cheminformatics and molecular docking approaches were used to identify the pharmacological effects of phytocompounds and their interactions with prognostic genes, respectively. Further, this study is the touchstone for the application of herbal medicines in multimodal therapy for cachexia. Overall, in this pilot study, potential phytocompounds are identified for CAC therapy using integrated omics and network pharmacology approaches; however, in vitro and in vivo experiments are needed to confirm the results.

## Figures and Tables

**Table 1:** List of selected medicinal plants and phytocompounds with their abbreviations.

S. No	Phytocompounds		Abbreviation	References
1.	*Coptis chinensis*	Berberine Palmatine Coptisine	BER PAL COP	[33,34,35,36]
2.	*Atractylodesmacrocephala*	Atractylenolide I Taraxeryl acetate Lupeol	ATA TAR LUP	[37,38]
3.	*Panax ginseng* C. A. Meyer	Ginsenosides Ginseng Tetrapeptide	GSN GST	[39,40]
4.	*Zizyphus jujuba*	Lotusine B Jujubogenin Jujuboside A	LTB JJG JJA	[41,42,43]
5.	*Coix lacryma-jobi*	Coixenolide Βetasitosterol Caffeic acid Naringenin	CXL BST CFA NGN	[44,45]
6.	*Trigonella foenum-graecum* L.	Trigonelline Gentianine Quercetin	TGL GTN QRC	[46,47,48]
7.	*Pisum sativum*	Gallicacid Epicatechin gallate Kaempferol	GLA ECG KPF	[49,50]
8.	*Zingiber officinale*	6-gingerol Zingerone Citral	GGL ZGN CTL	[51,52]
9.	*Artemisia japonica* Thunb.	Beta-amyrin Β-sitosterol 7,8- dimethoxycoumarin	BMR BSL DMC	[53]
10.	*Xanthium strumarium* L.	Xanthinosin Caffeic acid Xanthanol	XNS CFA XNL	[54,55]
11.	*Chrysanthemum morifolium* Ramatuelle	Apigetrin Astragaloside Chlorogenic acid Kaemferol-3-O-rutinoside	AGT AGS CGA KFR	[56,57]
12.	*Carthamus tinctorius* L.	Carthamin Hydroxysafflor yellow A	CRN HYA	[58]

**Table 2:** List of phytocompounds and their targeted unique genes related to CAC.

S. No	Phytocompounds	Genes
1	ATA, LUP, NGN, GGL	PPARG
2	PAL, ATA, TAR, JJA, CFA, ECG, GGL, ZGN, HYA	STAT3
3	GST, GLA	ADRB2
4	GST	SIRT1
5	CXL	NLRP
6	NGN, CGA	IGFBP3
7	ECG, AGA, AGT, KFR	TNF
8	CFA, GTN, XNS	TLR4
9	PAL, JJA, CFA, NGN, TGL, QRC, ECG, KPF, CTL, XNX, AGA, AGT, CGA, KFR	MMP9
10	TAR, JJG, JJA, BST, CFA, TGL, GTN, GGL, ZGN, BMR, XNS, XNL,	NOS2
11	BER, TAR	QPCT
12	BER, PAL, COP, TGL, CGA, KFR, CRN, HYA	IKBKB
13	COP	TUBB3
14	COP, LTB, QRC, AGS, KFR	MYLK
15	GSN, GST, JJA, CXL, CFA, GTN, QRC, KPF, CTL, BMR, DMA, XNS, XNL, AGS, KFR, ATA	F2
16	TAR, JJA, BST, GGL, BMR, XNL	NR3C1
17	GSN, TGL, BMR, XNL	CCR2
18	GSN, JJA, CXL, BST, BMR, XNS	HSD11B2
19	LUP, GST, CFA, CTL, DMC, XNS, CGA,	ACE
20	LUP, GST, LTB, JJG, NGN, GTN, QRC, KPF, CTL, GGL, BMR, DMC, XNS, AGT, AGS, KFR, HYA	GSK3B
21	GST, CXL	CAPN1
22	GST	SLC5A1
23	LTB	BIRC3
24	JJG	MAP3K14
25	CFA	NGFR
26	CFA	RELA
27	NGN, GLA, ECG, AGT, AGS, KFR,	SERPINE1
28	TGL	HDAC3
29	XNL	EIF2AK3
30	AGT, AGS, KFR	PLG
31	AGT, AGS, KFR	TP53
32	CGA	SELP

**Table 3:** Pharmacological properties of selected phytocompounds.

S. No	Compounds	GPCR Ligand (GPCR)	Kinase Inhibitor (Ki)	Nuclear Receptor Ligand (Ncr)	Protease Inhibitor (Pi)	Enzyme Inhibitor (Ei)	No. of Violations (nVio)
1.	BER	−0.11	−0.27	−0.78	−0.35	0.82	1
2.	PAL	−0.10	−0.22	−0.65	−0.29	0.81	1
3.	COP	−0.06	−0.22	−0.82	−0.33	0.89	1
4.	ATA	−0.63	−0.72	0.27	−0.28	0.50	0
5.	TAR	0.10	−0.31	0.44	−0.04	0.41	1
6.	LUP	0.27	−0.42	0.85	0.15	0.52	1
7.	GSN	0.28	−0.28	0.71	0.12	0.65	1
8.	GST	0.49	−0.10	−0.08	0.95	0.41	2
9.	LTB	−0.01	−0.53	−0.68	0.44	−0.35	1
10.	JJG	0.22	−0.31	0.79	0.06	0.67	1
11.	JJA	−3.83	−3.92	−3.87	−3.8	−3.79	3
12.	CXL	−0.02	−0.25	−0.14	0.04	−0.07	2
13.	BST	0.14	−0.51	0.73	0.07	0.51	1
14.	CFA	−0.48	−0.81	−0.10	−0.79	−0.09	0
15.	NGN	0.03	−0.26	0.42	−0.12	0.21	0
16.	TGL	−0.82	−1.75	−3.35	−1.05	−0.09	0
17.	GTN	−0.53	−0.58	−0.81	−0.92	−0.09	0
18.	QRC	−0.06	0.28	0.36	−0.25	0.28	0
19.	GLA	−0.77	−0.88	−0.52	−0.94	−0.17	0
20.	ECG	0.17	0.05	0.34	0.13	0.25	1
21.	KPF	−0.10	0.21	0.32	−0.27	0.26	0
22.	GGL	0.16	−0.33	0.20	0.15	0.38	0
23.	ZGN	−0.58	−1.15	−0.59	−0.72	−0.07	0
24.	CTL	−0.86	−1.29	−0.42	−0.57	0.02	0
25.	BMR	0.22	−0.31	0.67	0.11	0.56	1
26.	DMC	−0.83	−0.76	−0.77	−0.95	−0.08	0
27.	XNS	0.00	−0.85	0.96	−0.31	0.68	0
28.	XNL	−0.31	−0.34	−0.05	−0.54	0.10	0
29.	AGT	0.10	0.14	0.31	0.02	0.43	1
30.	AGS	−0.14	−0.28	−0.42	−0.15	−0.05	3
31.	CGA	0.29	−0.00	0.74	0.27	0.62	1
32.	KFR	−0.01	−0.09	−0.17	−0.04	0.18	3
33.	CRN	−3.36	−3.64	−3.59	−2.83	−3.35	3
34.	HYA	−0.11	−0.32	−0.06	−0.02	0.03	3

**Table 4:** Molecular docking results of selected phytocompounds and unique genes.

Phytocompound (Ligand)	Gene (Target)	Binding Energy ∆G (kcal/mol)
Apigetrin (AGT)	SERPINE1	−8.54
	GSK3B	−9.36
	PLG	−8.14
Naringenin (NGN)	SERPINE1	−7.02
	GSK3B	−7.04
Berberine (BER)	IKBKB	−6.11
	QPCT	−6.54

## Data Availability

All data generated or analyzed during this study are included in this article and its Supplementary Files.

## Supplementary Materials

The following supporting information can be downloaded at: https://www.mdpi.com/article/10.3390/ijms25010156/s1.
