# Supplementary material for: Unlocking Prognostic Genes and Multi-Targeted Therapeutic Bioactives from Herbal Medicines to Combat Cancer-Associated Cachexia: A Transcriptomics and Network Pharmacology Approach
Source: Int J Mol Sci. 2023 Dec 21;25(1):156. doi: 10.3390/ijms25010156 (PMC10778733; doi:10.3390/ijms25010156)
Supplement: Supplementary file 1 [file ijms-25-00156-s001.zip › ijms-2720633-supplementary.pdf]

Table S1. List of phytocompounds and their canonical SMILES used in this study.

S. No.	Compound Name	Abbreviation	Canonical SMILES
1	Berberine	BER	COC1=C(C2=C[N+]3=C(C=C2C=C1)C4=CC5=C(C=C4CC3)OCO5)OC
2	Palmatine	PAL	COC1=C(C2=C[N+]3=C(C=C2C=C1)C4=CC(=C(C=C4CC3)OC)OC)OC
3	Coptisine	COP	C1C[N+]2=C(C=C3C=CC4=C(C3=C2)OCO4)C5=CC6=C(C=C51)OCO6
4	Atractylenolide I	ATA	CC1=C2CC3C(=C)CCCC3(C=C2OC1=O)C
5	Taraxeryl acetate	TAR	CC(=O)OC1CCC2(C(C1(C)C)CCC3(C2CCC4(C3=CCC5(C4CC(CC5)(C)C)C)C)C)C
6	Lupeol	LUP	CC(=C)C1CCC2(C1C3CCC4C5(CCC(C(C5CCC4(C3(CC2)C)C)(C)C)O)C)C
7	Ginsenosides	GSN	CC(=CCCC(C)(C1CCC2(C1CCC3C2(CCC4C3(CCC(C4(C)C)O)C)C)C)O)C
8	Ginseng Tetrapeptide	GST	CC(C)C(C(=O)NC(CCC(=O)NC(CCCN=C(N)N)C(=O)NCC(=O)O)C(=O)O)N
9	Lotusine B	LTB	CCC(C)C1C(=O)NC=CC2=CC=C(C=C2)OC3CCN(C3C(=O)N1)C(=O)C(CC4=CC=CC=C4)NC(=O)C(CC(C)C)N(C)C
10	Jujubogenin	JJG	CC(=CC1CC(C2C3CCC4C5(CCC(C(C5CCC4(C36CC2(O1)OC6)C)(C)C)O)C)(C)O)C
11	Jujuboside A	JJA	CC1C(C(C(C(O1)OC2C(C(COC2OC3CCC4(C5CCC6C7C(CC(OC78CC6(C5(CCC4C3(C)C)C)CO8)C=C(C)C)(C)O)C)O)OC9C(C(C(C(O9)COC1C(C(C(C(O1)CO)O)O)O)O)O)O)OC1C(C(C(CO1)O)O)O)O)O)O
12	Coixenolide	CXL	CCCCCCC=CCCCCCCCCCC(=O)OC(C)C(C)OC(=O)CCCCCCCC=CCCCCCC
13	Betasitosterol	BST	CCC(CCC(C)C1CCC2C1(CCC3C2CC=C4C3(CCC(C4)O)C)C)C(C)C
14	Caffeic acid	CFA	C1=CC(=C(C=C1C=CC(=O)O)O)O
15	Naringenin	NGN	C1C(OC2=CC(=CC(=C2C1=O)O)O)C3=CC=C(C=C3)O
16	Trigonelline	TGL	C[N+]1=CC=CC(=C1)C(=O)[O-]
17	Gentianine	GTN	C=CC1=CN=CC2=C1CCOC2=O
18	Quercetin	QRC	C1=CC(=C(C=C1C2=C(C(=O)C3=C(C=C(C=C3O2)O)O)O)O)O
19	Gallicacid	GLA	C1=C(C=C(C(=C1O)O)O)C(=O)O
20	Epicatechin gallate	ECG	C1C(C(OC2=CC(=CC(=C21)O)O)C3=CC(=C(C=C3)O)O)OC(=O)C4=CC(=C(C(=C4)O)O)O

21	Kaempferol	KPF	C1=CC(=CC=C1C2=C(C(=O)C3=C(C=C(C=C3O2)O)O)O)O
22	6-Gingerol	GGL	CCCCCC(CC(=O)CCC1=CC(=C(C=C1)O)OC)O
23	Zingerone	ZGN	CC(=O)CCC1=CC(=C(C=C1)O)OC
24	Citral	CTL	CC(=CCCC(=CC=O)C)C
25	Beta-Amyrin	BMR	CC1(CCC2(CCC3(C(=CCC4C3(CCC5C4(CCC(C5(C)C)O)C)C)C2C1)C)C)C
26	7,8-dimethoxycoumarin	DMC	COC1=C(C2=C(C=C1)C=CC(=O)O2)OC
27	Xanthinosin	XNS	CC1CC2C(CC=C1CCC(=O)C)C(=C)C(=O)O2
28	Xanthanol	XNL	C1=CC=C2C(=C1)C(C3=CC=CC=C3O2)O
29	Apigetrin	AGT	C1=CC(=CC=C1C2=CC(=O)C3=C(C=C(C=C3O2)OC4C(C(C(C(O4)CO)O)O)O)O)O
30	Astragaloside	AGS	COC1=C(C=CC(=C1)C2=C(C(=O)C3=C(C=C(C=C3O2)O)O)OC4C(C(C(C(O4)COC5C(C(C(C(O5)CO)O)O)O)O)O)O)O
31	Chlorogenic acid	CGA	C1C(C(C(CC1(C(=O)O)O)OC(=O)C=CC2=CC(=C(C=C2)O)O)O)O
32	Kaemferol-3-O-rutinoside	KFR	CC1C(C(C(C(O1)OCC2C(C(C(C(O2)OC3=C(OC4=CC(=CC(=C4C3=O)O)O)C5=CC=C(C=C5)O)O)O)O)O)O)O
33	Carthamin	CRN	C1=CC(=CC=C1C=CC(=O)C2=C(C(C(=C(C2=O)C=C3C(=O)C(=C(C(C3=O)(C4C(C(C(C(O4)CO)O)O)O)O)O)C(=O)C=CC5=CC=C(C=C5)O)O)(C6C(C(C(C(O6)CO)O)O)O)O)O)O
34	Hydroxysafflor yellow A	HYA	C1=CC(=CC=C1C=CC(=C2C(=C(C(=O)C(C2=O)(C3C(C(C(C(O3)CO)O)O)O)O)C4C(C(C(C(O4)CO)O)O)O)O)O)O

Table S2. KEGG pathway enrichment analysis of unique genes and their involvement in CAC-related metabolic pathways.

Pathway.ID	Pathway Description	p.Val	FDR	Genes
KEGG:05417	Lipid and atherosclerosis	5.04E-10	5.04E-10	IKBKB,STAT3,MMP9,PPARG,GSK3B,NLRP3,TLR4,RELA,TNF,EIF2AK3,TP53,SELP
KEGG:04210	Apoptosis	1.85735E-06	1.85735E-06	IKBKB,CAPN1,BIRC3,MAP3K14,RELA,TNF,EIF2AK3,TP53
KEGG:05142	Chagas disease	5.29392E-06	5.29392E-06	IKBKB,NOS2,ACE,TLR4,RELA,SERPINE1,TNF
KEGG:05171	Coronavirus disease - COVID-19	8.32947E-06	8.32947E-06	IKBKB,STAT3,F2,ACE,NLRP3,TLR4,RELA,TNF,SELP
KEGG:05145	Toxoplasmosis	8.98276E-06	8.98276E-06	IKBKB,STAT3,NOS2,BIRC3,TLR4,RELA,TNF
KEGG:05162	Measles	4.75766E-05	4.75766E-05	IKBKB,STAT3,GSK3B,TLR4,RELA,EIF2AK3,TP53
KEGG:04936	Alcoholic liver disease	5.49983E-05	5.49983E-05	IKBKB,GSK3B,SIRT1,MAP3K14,TLR4,RELA,TNF
KEGG:05160	Hepatitis C	0.00010839	0.00010839	IKBKB,STAT3,GSK3B,RELA,TNF,EIF2AK3,TP53
KEGG:05161	Hepatitis B	0.000128361	0.000128361	IKBKB,STAT3,MMP9,TLR4,RELA,TNF,TP53
KEGG:04064	NF-kappa B signaling pathway	0.000133846	0.000133846	IKBKB,BIRC3,MAP3K14,TLR4,RELA,TNF
KEGG:05131	Shigellosis	0.000187411	0.000187411	IKBKB,GSK3B,CAPN1,NLRP3,TLR4,RELA,TNF,TP53
KEGG:04668	TNF signaling pathway	0.000243938	0.000243938	IKBKB,MMP9,BIRC3,MAP3K14,RELA,TNF
KEGG:05202	Transcriptional misregulation in cancer	0.000414985	0.000414985	MMP9,PPARG,BIRC3,NGFR,RELA,IGFBP3,TP53
KEGG:05130	Pathogenic Escherichia coli infection	0.000475524	0.000475524	IKBKB,TUBB3,F2,NLRP3,TLR4,RELA,TNF
KEGG:05133	Pertussis	0.000614228	0.000614228	NOS2,NLRP3,TLR4,RELA,TNF
KEGG:05135	Yersinia infection	0.000713926	0.000713926	IKBKB,GSK3B,NLRP3,TLR4,RELA,TNF
KEGG:05200	Pathways in cancer	0.001097739	0.001097739	IKBKB,STAT3,MMP9,PPARG,F2,NOS2,GSK3B,BIRC3,RELA,TP53
KEGG:04932	Non-alcoholic fatty liver disease	0.001454047	0.001454047	IKBKB,PPARG,GSK3B,RELA,TNF,EIF2AK3
KEGG:04657	IL-17 signaling pathway	0.001485521	0.001485521	IKBKB,MMP9,GSK3B,RELA,TNF
KEGG:04218	Cellular senescence	0.001564786	0.001564786	CAPN1,SIRT1,RELA,SERPINE1,IGFBP3,TP53
KEGG:05222	Small cell lung cancer	0.001566713	0.001566713	IKBKB,NOS2,BIRC3,RELA,TP53
KEGG:04217	Necroptosis	0.001743585	0.001743585	STAT3,CAPN1,BIRC3,NLRP3,TLR4,TNF
KEGG:05215	Prostate cancer	0.002025969	0.002025969	IKBKB,MMP9,GSK3B,RELA,TP53
KEGG:05132	Salmonella infection	0.002262994	0.002262994	IKBKB,TUBB3,BIRC3,NLRP3,TLR4,RELA,TNF
KEGG:05164	Influenza A	0.002381209	0.002381209	IKBKB,NLRP3,TLR4,RELA,TNF,PLG
KEGG:04660	T cell receptor signaling pathway	0.002708325	0.002708325	IKBKB,GSK3B,MAP3K14,RELA,TNF

KEGG:04625	C-type lectin receptor signaling pathway	0.002837532	0.002837532	IKBKB,MAP3K14,NLRP3,RELA,TNF
KEGG:04931	Insulin resistance	0.003402919	0.003402919	IKBKB,STAT3,GSK3B,RELA,TNF
KEGG:04066	HIF-1 signaling pathway	0.003557021	0.003557021	STAT3,NOS2,TLR4,RELA,SERPINE1
KEGG:04621	NOD-like receptor signaling pathway	0.003734966	0.003734966	IKBKB,BIRC3,NLRP3,TLR4,RELA,TNF
KEGG:05010	Alzheimer disease	0.004654905	0.004654905	IKBKB,TUBB3,NOS2,GSK3B,CAPN1,RELA,TNF,EIF2AK3
KEGG:04722	Neurotrophin signaling pathway	0.005200676	0.005200676	IKBKB,GSK3B,NGFR,RELA,TP53
KEGG:05321	Inflammatory bowel disease	0.005804451	0.005804451	STAT3,TLR4,RELA,TNF
KEGG:05169	Epstein-Barr virus infection	0.005975193	0.005975193	IKBKB,STAT3,MAP3K14,RELA,TNF,TP53
KEGG:04380	Osteoclast differentiation	0.006842309	0.006842309	IKBKB,PPARG,MAP3K14,RELA,TNF
KEGG:04920	Adipocytokine signaling pathway	0.008829943	0.008829943	IKBKB,STAT3,RELA,TNF
KEGG:05140	Leishmaniasis	0.010424787	0.010424787	NOS2,TLR4,RELA,TNF
KEGG:05418	Fluid shear stress and atherosclerosis	0.010922754	0.010922754	IKBKB,MMP9,RELA,TNF,TP53
KEGG:05163	Human cytomegalovirus infection	0.011510504	0.011510504	IKBKB,STAT3,GSK3B,RELA,TNF,TP53
KEGG:05212	Pancreatic cancer	0.012863415	0.012863415	IKBKB,STAT3,RELA,TP53
KEGG:01523	Antifolate resistance	0.013389849	0.013389849	IKBKB,RELA,TNF
KEGG:04211	Longevity regulating pathway	0.023651662	0.023651662	PPARG,SIRT1,RELA,TP53
KEGG:05235	PD-L1 expression and PD-1 checkpoint pathway in cancer	0.023651662	0.023651662	IKBKB,STAT3,TLR4,RELA
KEGG:04933	AGE-RAGE signaling pathway in diabetic complications	0.036893696	0.036893696	STAT3,RELA,SERPINE1,TNF
KEGG:05146	Amoebiasis	0.038313492	0.038313492	NOS2,TLR4,RELA,TNF
KEGG:04620	Toll-like receptor signaling pathway	0.03977185	0.03977185	IKBKB,TLR4,RELA,TNF
KEGG:04062	Chemokine signaling pathway	0.047993483	0.047993483	IKBKB,STAT3,CCR2,GSK3B,RELA

Table S3. List of cachexia-related genes used in this study.

S. No	Gene	Reference
1	mTORC1	Setiawan et al. 2023
2	mTORC2	
3	MYOD1	
4	UCP1	
5	IGF1	
6	AKT	
7	JUP	
8	BMP	
9	BMP7	
10	MSTN	
11	ACVR1	
12	SMAD2	
13	SMAD3	
14	NFKB1	
15	MAP3K14	
16	TNF	
17	STAT3	
18	IL6	
19	BNIP3	
20	LC3B	
21	FOXO3	
22	ATG7	
23	PI3K	
24	CAPN1	
25	KCTD7	
26	PIF	

27	TRIM63	
28	SLC39A4	
29	PDK4	
30	SDF1	
31	CEBPA	
32	CXCR4	
33	EIF2AK3	
34	COL1A1	Narasimhan et al. 2021
35	HMGCS2	
36	COL3A1	
37	ASPN	
38	DDN	
39	TMEM8C	
40	CHI3L1	
41	LOC653513	
42	MXRA5	
43	SAA2	
44	THY1	
45	CPXM1	
46	HP	
47	SFRP4	
48	SLC5A1	
49	MMP9	
50	TYRP1	
51	NOV	
52	COL1A2	
53	ECEL1	
54	KRT80	
55	CCL19	

56	COL5A1
57	SFRP2
58	SERPINE1
59	FZD10
60	GLT1D1
61	CRABP2
62	STMN2
63	CCL3
64	TPPP3
65	TMEM119
66	LUM
67	FXYD4
68	SPON1
69	TNC
70	ELN
71	SLAMF8
72	CILP2
73	CCL13
74	CCDC80
75	OGN
76	MOXD1
77	CD69
78	ERBB3
79	LGI2
80	IGJ
81	CAMSAP3
82	CKB
83	COL5A2
84	MSMP

85	TTC39A
86	MGC39372
87	C2
88	CD163L1
89	ZNF556
90	CELSR1
91	BHLHE22
92	MYH3
93	CTHRC1
94	TUBB3
95	KAZALD1
96	IGLL5
97	ESM1
98	MYBPH
99	COL14A1
100	TNFAIP6
101	FAIM2
102	PHOSPHO1
103	VASH2
104	SMIM5
105	CH25H
106	GDF10
107	CERCAM
108	FNDC1
109	CD200R1
110	CYS1
111	SHANK1
112	MYL5
113	CRISPLD1

114	FAM180B	
115	CRMP1	
116	PDE6G	
117	LTK	
118	GADD45G	
119	ABLIM1	
120	TNFRSF18	
121	NLRP3	
122	ARHGDIG	
123	CASKIN1	
124	IGSF10	
125	SIGLEC9	
126	THBS4	
127	ADAMTS2	
128	EMB	
129	RASD1	
130	MMP11	
131	LBP	
132	ITGB8	
133	LRRC17	
134	FPR3	
135	NGFR	
136	PRG4	
137	CCR2	
138	INHBE	
139	ABCC6	
140	SLC45A3	
141	OXCT2	
142	FZD2	

143	FAM57B	
144	IL4I1	
145	IL7R	
146	CCL4	
147	HSD11B2	
148	RHOH	
149	TNFAIP8L3	
150	PMAIP1	
151	LILRB4	
152	PNMA2	
153	PITPNM1	
154	KDELR3	
155	GPR183	
156	DIO2	
157	TMEM59L	
158	CCL2	
159	GNA15	
160	SOX3	
161	CILP	
162	BRSK1	
163	PRUNE2	
164	QPCT	
165	CYP4F11	
166	IFITM10	
167	CERKL	
168	ST6GALNAC2	
169	FOXI2	
170	BICD1	
171	ASPHD2	

172	ABO		
173	SVOP		
174	OSM		
175	VCAN		
176	OMD		
177	SOCS3		
178	TNFRSF9		
179	MYLK		
180	PAPLN		
181	VIPR2		
182	ITGA11		
183	SELP	Tan et al. 2011	
184	LTA		
185	IL-1B		
186	ACE		
187	LPL		
188	RETN		
189	ADIPOQ		
190	Akt		
192	Ap1		
193	ERK1		
194	ERK2		
195	IFNG		
196	IGFBP3		
197	IgG		
198	IL12		
199	LDL		
200	LEP		
201	Mapk		

202	NFkB
204	MAPK
205	PDGFB
206	PPARG
207	Sod
208	TLR2
209	TLR4
210	Vegf
211	ADRB2
212	BIRC3
213	BTG2
214	CAMP
215	CREBBP
216	CRP
217	CSF1
218	F2
219	GSK3B
220	HDAC3
221	HGF
222	HMGB1
223	HSPD1
224	IKBKB
225	IL17A
226	IL6R
227	IRS1
228	LTF
229	NFIC
230	NOS2
231	NR3C1

232	PLD1	
233	PLG	
234	RELA	
235	SIRT1	
236	TNFSF12	
237	TP53	
238	TSC22D3	
